# Exploring the Relationship Between the CFPB Financial Well-Being Score and the Elder Index

**DOI:** 10.1093/geroni/igaa057.2599

**Published:** 2020-12-16

**Authors:** Jan Mutchler

**Affiliations:** University of Massachusetts Boston, Boston, Massachusetts, United States

## Abstract

The Elder Index is a cost of living indicator that measures the income older adults need to meet their living expenses while staying independent in the community, calculated on a county-by-county basis for the United States. Analyses based on the Elder Index show that a large segment of the age 65+ population has incomes below the Index, reflecting a level of insecurity that is considerably higher than suggested by the poverty rate. Moreover, comparison of the Elder Index to household income illustrates differences across states in the extent to which incomes cover the cost of necessary expenditures. In this paper we explore how cost of living contributes to subjective financial security among older people, as measured by the CFPB Financial Well-Being Score, using a data match of the Understanding America Study with the Elder Index. Results document this association, offering insight to spatial patterns of financial insecurity in later life.

